# Insight into the role of Sho1 in high-hydrostatic pressure and oxidative stress adaptation of the hadal fungus *Aspergillus sydowii* DM1

**DOI:** 10.1128/aem.00898-26

**Published:** 2026-07-16

**Authors:** Hongfu Lai, Guangzhao Hu, Yukun Cui, Xi Yu

**Affiliations:** 1 Shanghai Engineering Research Center of Hadal Science and Technology, College of Oceanography and Ecological Science, Shanghai Ocean University 74595, Shanghai, China; Chalmers tekniska hogskola AB, Gothenburg, Sweden

**Keywords:** deep-sea fungi, high hydrostatic pressure, Sho1, HOG-MAPK pathway, oxidative stress, CRISPR–Cas9

## Abstract

Deep-sea extreme environments harbor microorganisms with unique adaptive mechanisms. Fungi play significant ecological roles in these ecosystems, yet the regulatory pathways governing their adaptation to extreme conditions remain poorly understood. Here, we investigated the hadal-derived fungus *Aspergillus sydowii* DM1 as a model strain. Using CRISPR–Cas9 technology, we constructed a *sho1* gene knockout mutant to examine its phenotypic and molecular responses to osmotic stress, oxidative stress, and high hydrostatic pressure (HHP). Our results showed that Sho1 contributed to cell wall integrity, was associated with changes in secondary metabolite profiles, and modulated the high-osmolarity glycerol (HOG) responses. Under osmotic stress, deletion of *sho1* enhanced colony growth and conidiation under moderate-to-high salinity, and was accompanied by altered asexual developmental progression and HOG-associated gene expression. Under oxidative stress, loss of Sho1 led to increased reactive oxygen species (ROS) accumulation, and reduced spore viability. Under HHP, deletion of *sho1* impaired early pressure-responsive signaling, increased ROS accumulation, and reduced spore germination during recovery. Comparative analysis of *A. sydowii* strains from different ecological niches further suggested that the deep-sea strain may possess a more coordinated pressure-response pattern associated with the Sho1–Hog1 pathway. Together, these findings indicate that Sho1 functions as an important upstream coordinator linking environmental sensing with osmotic adaptation, oxidative homeostasis, developmental regulation, and pressure-associated recovery in a hadal filamentous fungus, offering new insights into fungal signal transduction and adaptive evolution in extreme environments.

**IMPORTANCE:**

Deep-sea fungi live under extreme conditions, yet the signaling mechanisms that help them survive remain poorly understood. Using a hadal isolate of *Aspergillus sydowii*, we show that the membrane sensor Sho1 contributes to fungal responses to osmotic stress, oxidative stress, and high hydrostatic pressure. Loss of *sho1* altered growth, development, redox balance, and recovery after pressure exposure, indicating that this upstream signaling component coordinates multiple adaptive traits in a deep-sea fungus. These findings extend current knowledge of fungal stress signaling beyond model organisms and suggest that conserved signaling modules can be repurposed for life in the deep ocean. This study provides a useful genetic framework for understanding how fungi adapt to extreme marine environments.

## INTRODUCTION

The hadal ecosystem (generally referring to ocean depths greater than 6,000 m) is considered one of the most extreme ecosystems on Earth (1), characterized by low temperature, ultra-high hydrostatic pressure (HHP), oligotrophic conditions, and permanent darkness, which impose stringent constraints on microbial survival (2, 3). In recent years, with continuous advances in hadal sediment sampling technologies, a wide variety of cultivable microorganisms have been isolated from hadal sediments (4, 5), revealing that these extreme environments harbor abundant microbial life with metabolic activities and ecological functions far exceeding previous expectations. Among these microorganisms, eukaryotic microbes, such as fungi, protozoa, and algae, play important roles in hadal nitrogen cycling, organic matter degradation, heavy metal transformation, and the biosynthesis of secondary metabolites (SMs) (6–9). Recently, Li et al. isolated multiple piezotolerant filamentous fungi from hadal sediments and identified a total of 41 fungal strains capable of normal growth under pressures ranging from 20 to 60 MPa (10), highlighting the remarkable metabolic potential and adaptive capacity of hadal fungi in biogeochemical cycles. Among them, *Aspergillus sydowii*, one of the commonly occurring filamentous fungi in hadal ecosystems (11, 12), has been repeatedly isolated from hadal sediments such as those of the Mariana Trench and exhibits notable piezotolerance, halotolerance, and the capacity to produce bioactive secondary metabolites (13, 14). These characteristics of deep-sea fungi have further stimulated interest in investigating their physiological and molecular responses to diverse environmental stresses.

The extreme conditions of hadal ecosystems pose formidable challenges to life (15), and eukaryotic signaling pathways play crucial roles in adaptation to such environments, among which mitogen-activated protein kinase (MAPK) signaling pathways are particularly important (16, 17). The high-osmolarity glycerol (HOG) signaling cascade is highly conserved in fungi (18) and primarily functions in sensing and responding to environmental stresses such as osmotic and oxidative stress, thereby maintaining cellular homeostasis and enabling adaptation to multiple extreme conditions through the activation of downstream gene expression (19–21); it serves as a central pathway for fungal responses to hyperosmotic, oxidative, and cell wall stresses (13, 22). In yeast, the HOG pathway perceives external stimuli through the upstream two-component sensor Sln1 and the four-transmembrane protein Sho1 (23), which converge on the MAPKK Pbs2 to activate the Hog1 kinase, thereby regulating the expression of antioxidant enzymes, cell wall biosynthetic enzymes, and osmoprotectants (24–28). In *Aspergillus sydowii* DM1, our previous genomic and functional studies supported the presence of conserved HOG-associated signaling components and implicated this pathway in pressure-related stress responses (13, 14). However, direct experimental evidence for the roles of upstream HOG-associated genes in hadal-derived *Aspergillus* species remains limited.

Sho1 is considered one of the key upstream sensors of the fungal HOG signaling pathway, capable of rapidly activating the MAPK-HOG cascade through interactions between its SH3 domain and proteins such as Pbs2 and Ste11 (29), and also functioning as a scaffold protein linking multiple downstream response pathways involved in osmotic stress, oxidative stress, and cell wall homeostasis (30). Studies in model and pathogenic fungi have demonstrated that the biological functions of Sho1 exhibit pronounced species specificity and functional diversity. In *Botrytis cinerea*, *sho1* contributes to vegetative growth, conidiation, sclerotium formation, and responses to osmotic stress, and its deletion results in reduced growth and virulence (31). In systematic studies of the pathogenic fungus *Candida albicans*, Sho1 and its functionally redundant sensor Sln1 constitute the major pathways for Hog1 activation under hyperosmotic conditions, and deletion of *sho1* leads to heightened sensitivity to cell wall-perturbing agents and hydrogen peroxide, accompanied by impaired cell wall integrity and reduced oxidative stress tolerance (32). These observations indicate that, although Sho1 is a conserved upstream module, its physiological outputs are strongly shaped by species background and ecological context (33). Therefore, whether Sho1 plays a similar or expanded role in hadal-derived filamentous fungi, especially under pressure-relevant conditions, cannot be inferred directly from model systems and requires experimental validation in an appropriate deep-sea isolate.

In our previous studies, an efficient gene editing and functional validation platform was established in the hadal isolate *Aspergillus sydowii* DM1, and functional characterization of the *hog1* gene revealed the critical roles of the HOG system in energy metabolism and oxidative stress responses (13), providing a solid foundation for investigating upstream sensors of the HOG pathway in deep-sea adaptation. Importantly, DM1 represents a suitable system for this investigation because it is a hadal-derived and genetically tractable filamentous fungus with clear relevance to pressure-associated stress adaptation. Moreover, osmotic stress, oxidative stress, and HHP were selected in this study because they represent a canonical osmotic stimulus for HOG signaling, a closely related oxidative challenge, and the defining physical constraint of the hadal habitat, respectively. In the present study, we focused on representative HOG-associated genes, including the upstream sensors *sho1* and *sln1*, the MAPKKK *ste11*, and the MAPK *hog1*, and interpreted their transcriptional responses together with selected downstream or pathway-associated readouts linked to development, polarity, and oxidative defense. We successfully constructed and validated a *sho1* gene knockout strain of *Aspergillus sydowii* using the CRISPR–Cas9 system and systematically analyzed its phenotypic and molecular responses under osmotic stress, oxidative stress, and HHP, thereby testing whether this conserved upstream sensor contributes to stress coordination and high-pressure adaptation in a hadal fungal background.

## MATERIALS AND METHODS

### Sample collection, isolation, and culture conditions

Strains used in this study were obtained from four different ecological sources. The hadal sample, from which *Aspergillus sydowii* DM1 was isolated, was collected from the hadal zone of the Mariana Trench (142.2148°E, 11.3403°N) during the October 2021 expedition of the research vessel Challenge No. 1 (TS21). This sample was maintained in a pressure-retaining device and transferred under sterile conditions to minimize contamination and preserve the authenticity of hadal fungal isolation. For ecological-niche comparisons, the terrestrial strain JF6-3 was obtained from the China General Microbiological Culture Collection Center (CGMCC), the shallow-sea/coastal strain L-6 was isolated from sediment collected from the Luchaogang Beach area in Shanghai, China, and the deep-sea strain C-6 was isolated from a 3,000 m deep-sea sediment sample collected during the 2023 Western Pacific shared cruise. Sediment samples from the Luchaogang Beach area and the 3,000 m deep-sea site were stored in sterile plastic bags before fungal isolation. Fungal isolation and cultivation were performed as described in our previous study (34). Briefly, 100 μL of diluted sediment suspension was spread onto potato dextrose agar (PDA; potato infusion 20 g/L, glucose 20 g/L, NaCl 28 g/L, and agar 15 g/L) supplemented with ampicillin (100 μg/mL) and incubated at 28°C for 3–7 days. Emerging hyphae were transferred to fresh PDA, and subculturing was repeated at least three times to obtain pure cultures. All strains were subsequently maintained and cultured on PDA under the same laboratory conditions for comparative analyses.

### CRISPR–Cas9-mediated *sho1* gene knockout and mutant identification

In the hadal isolate *Aspergillus sydowii* DM1, the *sho1* gene was deleted using a pFC332-based CRISPR–Cas9 system. Two sgRNAs targeting the 5′ and 3′ ends of the *sho1* coding region were designed, and chimeric fragments F1 and F2 were generated by fusion PCR from pFC334 and pAK4, respectively. The *Streptococcus pyogenes* Cas9 ORF and a dual sgRNA self-cleaving cassette flanked by hammerhead (HH) and hepatitis delta virus (HDV) ribozymes were cloned into PacI-linearized pFC332 carrying AMA1 and a hygromycin resistance marker to generate the knockout plasmid (35). After amplification in *Escherichia coli* DH5α and confirmation by colony PCR and sequencing, the plasmid was introduced into DM1 protoplasts by PEG-mediated transformation (13). Transformants were selected on PDA containing hygromycin B (100 μg/mL), subcultured twice, and verified by PCR. During the original screening, three representative transformants were selected and designated Δ*sho1*-1, Δ*sho1*-2, and Δ*sho1*-3. PCR amplification using primers flanking the *sho1* locus showed the expected shortened fragments in these transformants compared with the wild-type (WT) strain. The validated mutant strains were stored in 15% glycerol at −80°C.

### Stress treatments and phenotypic analysis

To evaluate the role of *sho1* in hadal-derived *Aspergillus sydowii* DM1, WT and Δ*sho1* were compared under osmotic, oxidative, and cell wall stresses. For osmotic-stress assays, separate PDA media were prepared with defined NaCl concentrations (0, 0.5, 1.0, 1.5, and 2.0 M), rather than by adjusting salt content after plate preparation. NaCl was selected as the osmotic stressor because it is a standard and experimentally tractable salt for fungal HOG-related osmotic-stress assays and also allows direct comparison with our previous studies in DM1 (13, 14). Oxidative- and cell-wall-stress assays were performed on separately prepared PDA plates containing H_2_O_2_ (0, 2, 4, 6, and 8 mM) or Congo red (0, 250, 500, 800, and 1,000 μg/mL), respectively. These concentrations were checked and standardized throughout the revised manuscript. Purified spore suspensions were prepared as described previously (14) and adjusted to 1 × 10^6^ spores/mL. PDA plates containing NaCl (0, 0.5, 1, 1.5, and 2 M), H₂O₂ (0, 2, 4, 6, and 8 mM), or Congo red (0, 250, 500, 800, and 1,000 μg/mL) were inoculated centrally with 1 μL spore suspension and incubated at 28°C unless otherwise stated; H_2_O_2_ was added after cooling the medium to approximately 50°C. For oxidative-stress assays, different H_2_O_2_ concentration ranges were used according to the purpose and tolerance range of each assay. Colony growth on PDA plates was assessed at 0, 2, 4, 6, and 8 mM H_2_O_2_, because both WT and Δ*sho1* failed to grow normally at 10 mM H_2_O_2_ in preliminary assays. Spore germination was assessed at representative concentrations of 0, 4, and 6 mM H₂O₂. For ROS detection, spores were treated with 0, 4, 6, and 10 mM H_2_O_2_ to evaluate ROS accumulation under both moderate and extreme oxidative stress. For qPCR analysis, mycelial samples were exposed to 0, 6, and 10 mM H_2_O_2_ for 1 h before RNA extraction. Colony morphology, color, diameter, spore yield, and germination were recorded. Germination was assessed in corresponding liquid media after 8–12 h by counting 300–500 spores, and a germ tube length equal to or greater than the spore diameter was defined as germination (14). In addition to the 0.5–2.0 M NaCl plate assays used for growth and conidiation analyses, an additional 3.0 M NaCl condition was used in liquid medium to examine whether extreme osmotic stress altered the timing of asexual developmental stage transitions. This higher salinity was selected as a stringent osmotic challenge for developmental observation rather than for standard colony-diameter comparison. Microscopic observations were performed using an OLYMPUS BX53 microscope. All experiments included three biological replicates and at least three technical replicates per condition.

### Physiological and biochemical measurements

Intracellular reactive oxygen species (ROS) accumulation and cell wall chitin content were measured in WT and Δ*sho1*. ROS levels in spores exposed to salinity, H_2_O_2_, or HHP were determined using 2′,7′-dichlorodihydrofluorescein diacetate (DCFH-DA; Beyotime, Shanghai, China) according to the manufacturer’s instructions (13). Spore suspensions were mixed 1:1 with DCFH-DA to a final concentration of 10 μM, incubated in the dark at 25°C for 4 h, and fluorescence was measured with a microplate reader (TECAN, Shanghai, China) at 490/525 nm. Cell wall chitin content was determined by Calcofluor White (CW) staining (36). After 3 days of culture in potato dextrose broth (PDB), mycelia were washed three times with PBS, stained in 1 mL CW solution at 28°C for 20 min in the dark, rewashed, blotted dry, weighed, homogenized, and transferred into 96-well plates (200 μL per well; three biological replicates). Fluorescence intensity (RFU) was measured at 360/430 nm.

### Extraction of secondary metabolites, HPLC analysis, and antibacterial activity assay

Secondary metabolites of WT and Δ*sho1* were prepared under the same conditions. WT and Δ*sho1* were inoculated from 5 to 7-day PDA cultures into equal volumes of PDB and cultured at 28°C and 180 rpm for 10 days, a time point selected based on preliminary cultivation observations and laboratory experience, because visible changes in culture supernatant pigmentation became evident around this stage, suggesting sufficient accumulation of extracellular secondary metabolites for comparative extraction and analysis. After incubation, the cultures were filtered to remove the mycelia, and the resulting culture supernatants were collected for extraction. The culture supernatants were extracted three times with equal volumes of ethyl acetate, and the combined organic phases were concentrated under reduced pressure at 40°C. The crude extracts were dissolved in methanol to 100 mg mL^−1^ and filtered through a 0.22 μm membrane for HPLC and antibacterial assays. HPLC was performed on an Agilent 1260 Infinity II system with a Zorbax SB-C18 column (4.6 × 250 mm, 5 μm) at 40°C, using water (A) and methanol (B) for gradient elution at 1.0 mL min^−1^ and detection at 220 nm. Antibacterial activity was assessed by the filter paper disc diffusion method described by Xiao et al. (37) using *Staphylococcus aureus*, *Candida albicans*, *Escherichia coli*, *Enterococcus faecalis*, and *Chromobacterium violaceum*. Freshly activated suspensions of the indicator microorganisms (bacterial suspensions for the four bacterial indicators and a *Candida albicans* suspension for the fungal indicator) were spread onto LB agar plates. Discs containing test samples or methanol (negative control) were then applied. After incubation at 37°C for 12 h, inhibition zones were measured.

### HHP treatment

HHP treatment was conducted using a laboratory-scale high-pressure vessel. Prepared spore suspensions (1 × 10^6^ spores/mL) were aliquoted into sterile high-pressure bags, with 50 mL per bag, and hermetically sealed. During treatment, the bags were placed into the pressure chamber filled with distilled water. Based on comparisons with previous laboratory studies, the pressure gradients were set at 40, 60, and 80 MPa (14, 38), which were chosen as experimentally tractable pressure levels relevant to deep-sea and hadal conditions and to enable direct comparison with our previous pressure-response studies in hadal fungi. These pressures approximately correspond to hydrostatic pressures encountered at depths of 4,000, 6,000, and 8,000 m, respectively, thereby covering a pressure range from deep-sea to hadal-like conditions. After pressurization, samples were maintained at 28°C for 72 h. Upon completion of treatment, pressure was gradually released to atmospheric levels according to standard operating procedures. The treated spore suspensions were then collected for subsequent analyses, including spore germination assays, reactive oxygen species (ROS) detection, and colony recovery tests. To obtain mycelial samples for post-pressure transcriptional analysis, WT and Δ*sho1* strains were cultured in PDB with shaking for 3 days, and the resulting biomass was collected at the vegetative mycelial stage. Thus, the material used for RNA extraction represented mycelia rather than ungerminated spores or very early germlings. Pre-formed mycelia were used for transcriptional analysis in order to minimize the confounding effects of strain-dependent differences in spore germination and developmental timing under pressure, thereby allowing a more comparable assessment of pressure-responsive gene expression between WT and Δ*sho1*. The mycelia were harvested and subjected to HHP treatment as described above. Following treatment, the mycelial samples were immediately frozen in liquid nitrogen and processed with TRIzol reagent for subsequent RNA extraction.

### Gene expression analysis by quantitative real-time PCR (qRT-PCR)

Quantitative real-time PCR (qRT-PCR) was performed to assess the expression levels of key genes under different stress conditions. Total RNA extraction and cDNA synthesis were carried out according to the method described by Zhong et al. (14). Briefly, mycelial samples collected under various stress conditions were subjected to total RNA extraction using RNAiso Plus reagent (Takara, Japan). Genomic DNA was removed, and first-strand cDNA was synthesized using the PrimeScript RT reagent kit with gDNA Eraser (Takara). qRT-PCR amplification was performed on a QuantStudio real-time PCR system using SYBR Premix Ex Taq II (Tli RNaseH Plus). Relative expression levels of all target genes were calculated using the 2^−ΔΔCt^ method (39) and normalized to the reference gene GAPDH. Primer sequences used in this study are provided in Table S1.

## RESULTS

### Construction of the Δ*sho1* strain via CRISPR–Cas9

In this study, we targeted the *sho1* locus of *Aspergillus sydowii* DM1, which contains a 1,077-bp open reading frame (Fig. S1A). Based on its sequence similarity to known fungal Sho1 proteins, this locus was annotated as a Sho1 homolog. Sequence similarity and conserved-domain analyses further supported this annotation, with NCBI searches identifying fungal osmosensor SHO1 homologs and an SH3_Sho1p domain in the *sho1* sequence (Table S2). Fungal Sho1 proteins are generally membrane-associated upstream components of the HOG pathway and typically contain membrane-spanning regions and a conserved SH3 domain involved in downstream signaling interactions. These features support the annotation of Sho1 as a conserved HOG-associated upstream sensor in DM1. The Δ*sho1* strain of *Aspergillus sydowii* DM1 was generated using the CRISPR–Cas9 system (Fig. S1B). During the original knockout screening, three representative transformants were selected for PCR verification and designated Δ*sho1*-1, Δ*sho1*-2, and Δ*sho1*-3. PCR amplification using primers flanking the *sho1* locus produced the expected amplicon of ~1.6 kb in WT and shortened amplicons of ~0.7 kb in the Δ*sho1* transformants (Fig. S1C). Sequencing of the representative mutant further confirmed deletion of the 1,077-bp *sho1* coding region, corresponding to the complete predicted *sho1* ORF in DM1, with the flanking regions retained (Fig. S1D). Representative colony observations on PDA supplemented with 1 M NaCl showed that the three independent Δ*sho1* transformants displayed a consistent osmotic-stress-associated growth phenotype compared with WT (Fig. S1E). Δ*sho1*-1 was selected as the representative mutant for the detailed phenotypic and transcriptional analyses in this study.

### Sho1 is a key factor in maintaining cell wall integrity

**Fig 1:**
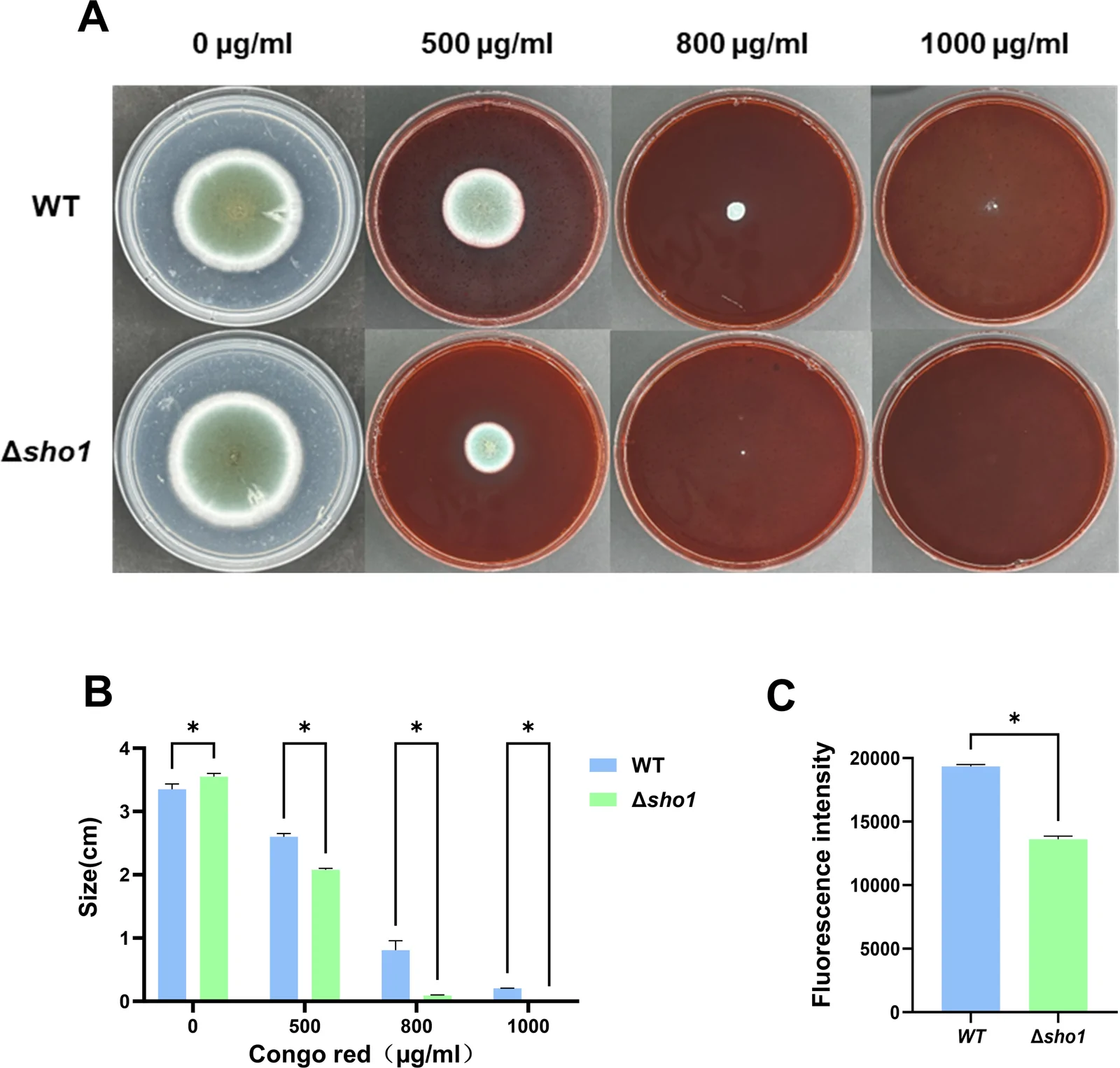
Cell wall stress sensitivity and chitin content analysis of WT and Δ*sho1* strains. (**A**) Colony morphology of the wild-type (WT) and Δ*sho1* strains on PDA plates supplemented with Congo red (0, 500, 800, and 1,000 µg/mL) after 7 days of incubation at 28°C. (**B**) Quantification of colony diameters of WT and Δ*sho1* strains under Congo red treatment after 7 days. (**C**) Chitin content of WT and Δ*sho1* strains under standard culture conditions assessed by Calcofluor White (CW) staining, shown as fluorescence intensity (RFU).The asterisk indicates a significant difference between WT and Δ*sho1* (*, *P* < 0.05).

To investigate the role of Sho1 in cell wall integrity, we examined the sensitivity of the wild-type (WT) and the Δ*sho1* to the cell wall–perturbing agent Congo red. Based on the quantitative data in Fig. 1B, Δ*sho1* already showed reduced colony growth relative to the WT under the control condition (0 μg/mL Congo red), and under Congo red treatment it consistently exhibited smaller colony diameters than the WT, with a significant difference already evident at 500 μg/mL (*P* < 0.05), indicating increased sensitivity to cell wall stress. This defect was further exacerbated at higher concentrations: Δ*sho1* exhibited severely restricted growth at 800 μg/mL and almost completely ceased growth at 1,000 μg/mL, while the WT still retained limited growth. To further assess cell wall–associated staining, Calcofluor White (CW) staining was performed. Under standard culture conditions, the fluorescence intensity of CW staining in the Δ*sho1* was significantly lower than that in the WT (Fig. 1C), reaching approximately 70% of the WT level (*P* < 0.05). These results suggest that Sho1 contributes to cell wall integrity in *Aspergillus sydowii* DM1.

### Sho1 acts as an osmosensor coordinating hyphal growth and conidiation

**Fig 2:**
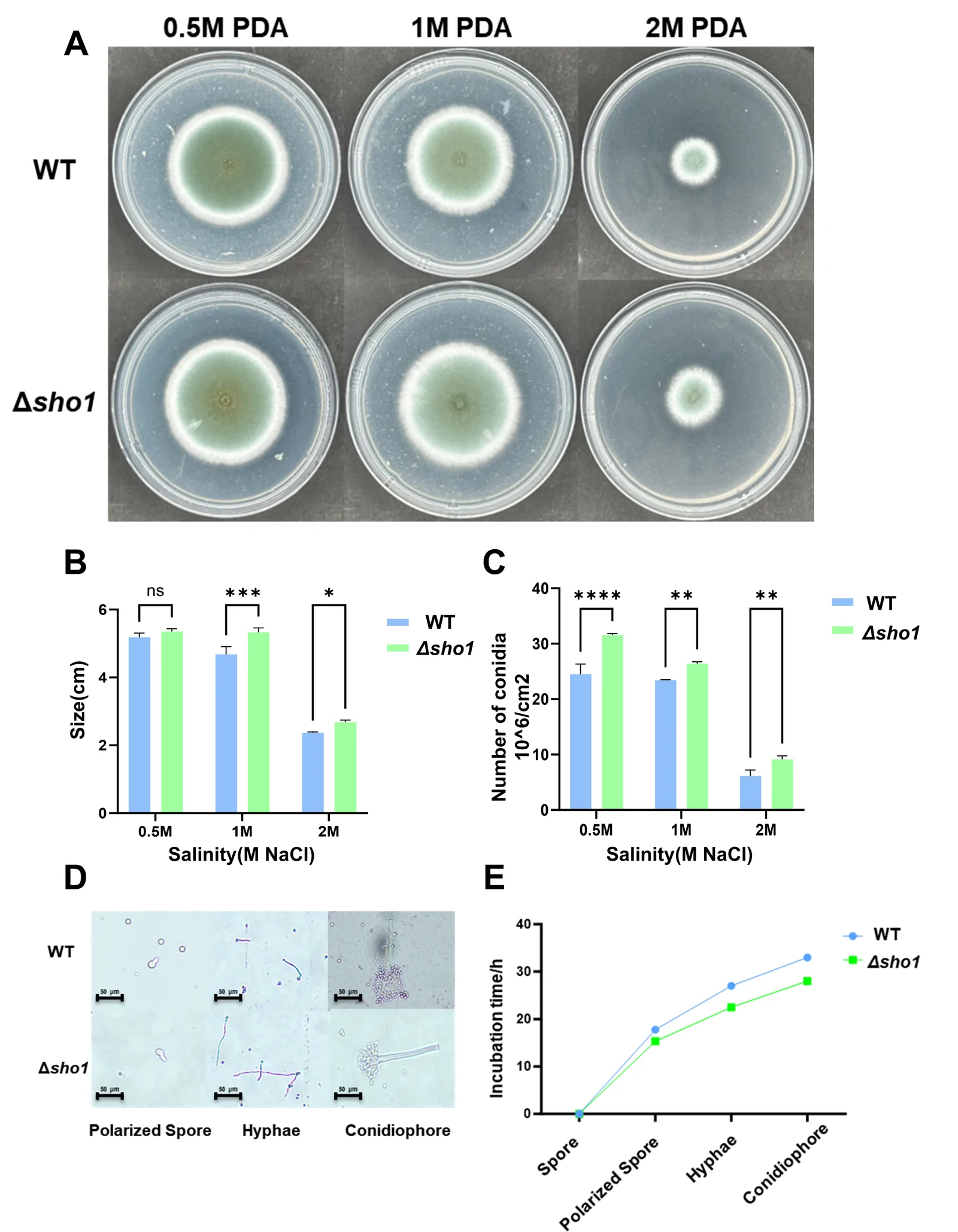
Phenotypic comparison of WT and Δ*sho1* under different salinity conditions. (**A**) Colony morphology of WT and Δ*sho1* strains on PDA plates containing 0.5, 1.0, and 2.0 M NaCl after 7 days of incubation at 28°C. (**B**) Colony diameter of WT and Δ*sho1* strains cultured under 0.5–2.0 M NaCl for 7 days. (**C**) Conidia production of WT and Δ*sho1* strains grown on PDA supplemented with 0.5–2.0 M NaCl for 7 days (10^6^ conidia/cm^2^). (**D**) Representative microscopic images showing asexual developmental stages of WT and Δ*sho1* under 3.0 M NaCl in liquid medium (spore, polarized spore, hyphae, and conidiophore). (**E**) Time-course analysis of the first appearance of each developmental stage under 3.0 M NaCl. Data represent the mean of three biological replicates. Significance labels indicate comparisons between WT and Δ*sho1* under the same NaCl condition: *, *P* < 0.05; **, *P* < 0.01; ***, *P* < 0.001; ****, *P* < 0.0001.

**Fig 3:**
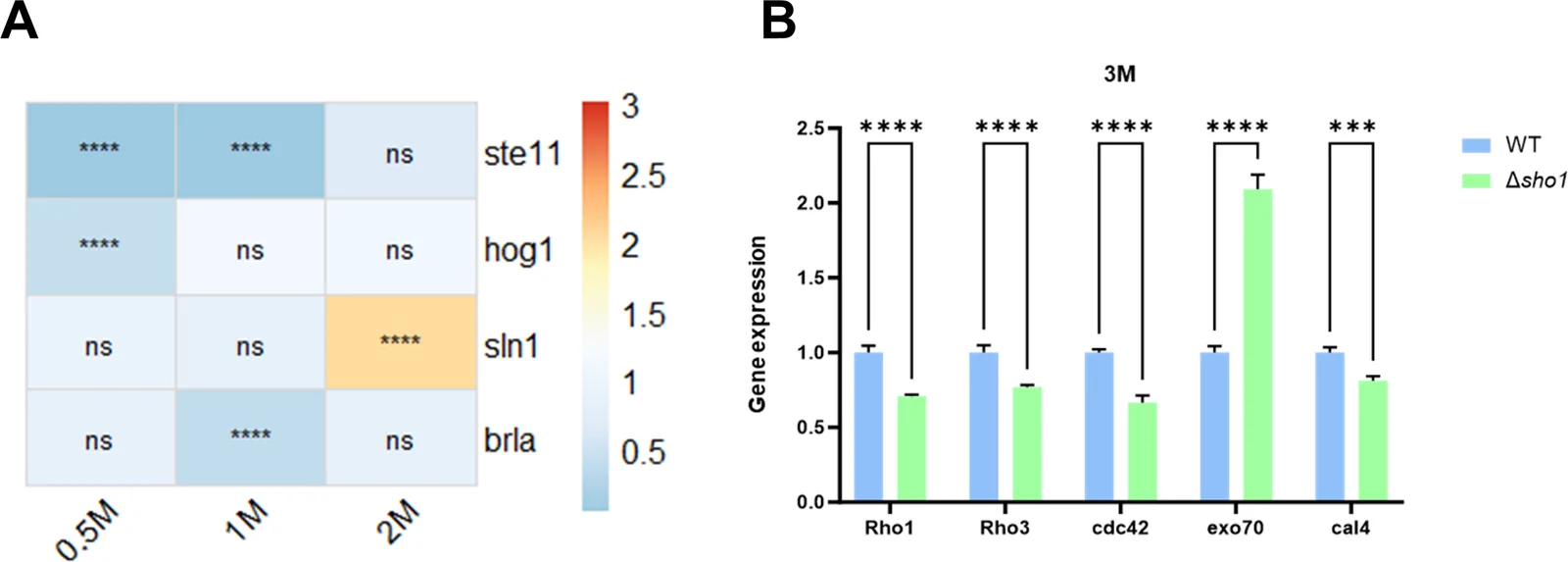
Summary of qPCR responses to NaCl stress in WT and Δ*sho1*. (**A**) Heatmap showing Δ*sho1*/WT expression fold changes (qPCR) of selected genes under 0.5–2.0 M NaCl, including HOG-associated signaling and asexual development–related genes (*ste11*, *hog1, sln1*, and *brlA*). (**B**) Expression responses of polarity/vesicle trafficking–related genes under 3.0 M NaCl. Values represent the ratio of mean expression levels in Δ*sho1* vs WT from three biological replicates. Significance of Δ*sho1* vs WT is indicated within each panel (ns, not significant; ***, *P* < 0.001; and ****, *P* < 0.0001). Full qPCR bar plots are provided as supplemental raw data (Fig. S2).

Osmotic stress is one of the primary challenges faced by microbes in marine environments. To investigate the role of *sho1* in this stress response in DM1, we compared growth and gene expression between WT and Δ*sho1* under different NaCl concentrations. Deletion of *sho1* was associated with enhanced colony growth under moderate to high salinity. When cultured on PDA plates supplemented with 0.5–2.0 M NaCl for 7 days, Δ*sho1* exhibited larger colony diameters than WT (Fig. 2A and B), with a significant difference observed at 1 M NaCl (5.33 cm vs 4.68 cm, *P* < 0.001). A similar trend was observed at 2 M NaCl (2.68 cm vs 2.37 cm, *P* < 0.05). Consistent with this growth phenotype, conidiation in Δ*sho1* was higher than that of WT across the tested salinity conditions (Fig. 2C), including at 1 M NaCl (26.40 × 10^6^ vs 23.41 × 10^6^ spores/cm^2^, *P* < 0.001). Because the 0.5–2.0 M NaCl plate assay was used primarily to evaluate colony growth and conidiation under moderate-to-high salinity, an additional 3.0 M NaCl condition was employed to examine whether a more stringent osmotic challenge affected the timing of asexual developmental transitions. Under extreme osmotic stress (3.0 M NaCl), representative microscopic images showed differences in asexual developmental progression between WT and Δ*sho1* (Fig. 2D). Consistent with these observations, a time-course analysis of the first appearance of each developmental stage indicated that Δ*sho1* reached the polarized spore, hyphae, and conidiophore stages earlier than WT under 3.0 M NaCl (Fig. 2E). Specifically, the first appearance times for polarized spores, hyphae, and conidiophores were ~15.3, ~23, and ~28 h in Δ*sho1*, compared with ~17.8, ~27, and ~33 h in WT, respectively (Fig. 2E). To interpret these phenotypic differences in a pathway context, qPCR assays were performed for representative HOG-associated genes, including the alternative upstream sensor *sln1*, the MAPKKK *ste11*, and the MAPK *hog1*, together with selected development- or polarity-related readouts such as *brlA*, *cdc42*, and *exo70* (Fig. 3). Under 0.5–2.0 M NaCl, Δ*sho1* showed markedly reduced expression of *ste11* and lower expression of *hog1* at 0.5 M, while *sln1* was significantly upregulated at 2.0 M (Fig. 3A), consistent with a salinity-dependent shift in upstream HOG-associated signaling. In addition, the asexual development regulator *brlA* was reduced at 1.0 M in Δ*sho1* (Fig. 3A). Under 3.0 M NaCl, the polarity regulator *cdc42* was downregulated, whereas the vesicle trafficking gene *exo70* was strongly upregulated in Δ*sho1* (Fig. 3B), which may be associated with the altered developmental dynamics observed under extreme salinity. Full qPCR bar plots are provided as supplemental raw data (Fig. S2). Together, these data indicate that under osmotic stress, deletion of *sho1* is associated with faster colony expansion and earlier asexual developmental progression, but with a rewired transcriptional pattern in core HOG-associated and polarity-related genes. This suggests that Sho1 contributes not only to osmotic sensing itself, but also to coordinating the balance between stress signaling and developmental timing in DM1.

### Sho1 contributes to oxidative stress resistance and spore viability by maintaining ROS homeostasis

**Fig 4:**
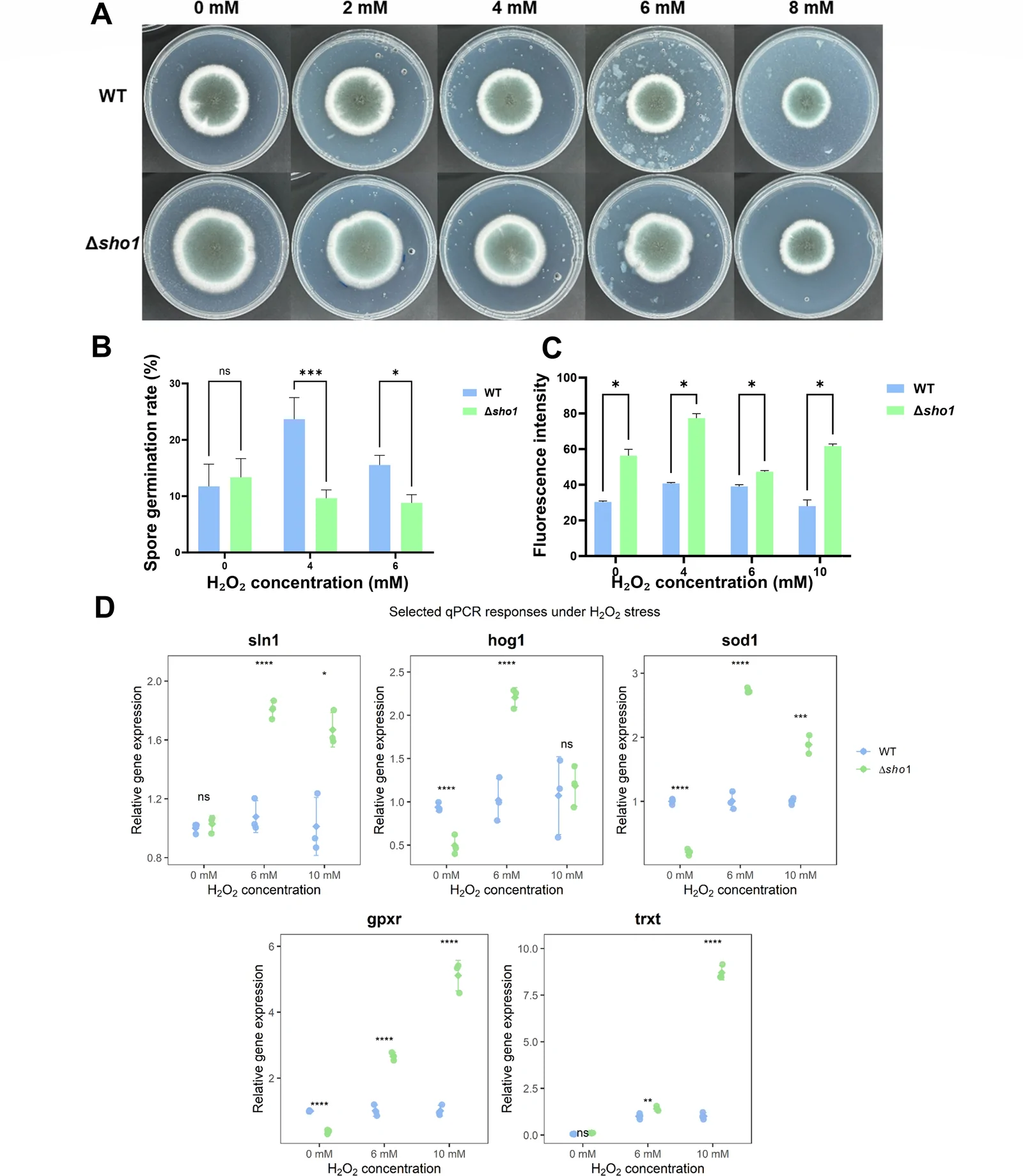
Deletion of Sho1 is associated with oxidative-stress responses and spore viability under H_2_O_2_ stress. (**A**) Representative colony morphologies of WT and Δ*sho1* strains on PDA plates supplemented with H_2_O_2_ (0, 2, 4, 6, and 8 mM) after incubation at 28°C for 7 days. At 10 mM H_2_O_2_, neither strain showed normal colony development; therefore, no representative colony image was included in this panel. (**B**) Spore germination rates of WT and Δ*sho1* strains under H_2_O_2_ treatment (0, 4, and 6 mM). (**C**) Intracellular ROS levels in spores of WT and Δ*sho1* under H_2_O_2_ treatment. (**D**) Selected qPCR responses of oxidative stress-related genes in WT and Δ*sho1* under H_2_O_2_ stress. Relative expression levels of *sln1*, *hog1*, *sod1*, *gpxr*, and *trxt* were measured in WT and Δ*sho1* after 1 h exposure to 0, 6, and 10 mM H_2_O_2_. Different H_2_O_2_ concentration ranges were used for colony growth, germination, ROS detection, and qPCR assays according to the purpose and tolerance range of each assay. Each dot represents one biological replicate; diamonds indicate the mean, and error bars indicate SD. Significance labels show comparisons between WT and Δ*sho1* within the same treatment condition (ns, not significant; *, *P* < 0.05; **, *P* < 0.01; ***, *P* < 0.001; and ****, *P* < 0.0001). Full qPCR bar plots for all tested genes are provided as supplemental raw data (Fig. S3B through D).

Given that osmotic stress and high hydrostatic pressure can disturb redox homeostasis and promote reactive oxygen species (ROS) accumulation, and that the HOG pathway is linked to oxidative stress defense, we further examined whether Sho1 is involved in oxidative stress responses. After 7 days of incubation on PDA plates containing 2–8 mM H_2_O_2_, the colony diameters of Δ*sho1* were larger than those of the WT (Fig. 4A; quantified in Fig. S3A). Specifically, at 2, 4, 6, and 8 mM H_2_O_2_, Δ*sho1* exhibited colony diameters of 4.95, 4.63, 4.50, and 3.48 cm, respectively, all of which were significantly greater than those of the WT (*P* < 0.05). At 10 mM H_2_O_2_, neither strain showed normal colony development, and representative colony images were therefore not included in Fig. 4A. These results suggest that deletion of *sho1* partially alleviates the inhibitory effect of H_2_O_2_ on colony growth. In contrast, spore germination assays indicated that Δ*sho1* was more sensitive to oxidative stress (Fig. 4B). Under 4 mM H_2_O_2_, the germination rate of Δ*sho1* decreased to 9.64%, significantly lower than that of the WT (23.67%, *P* < 0.001). A similar trend was observed at 6 mM H_2_O_2_, where the germination rate of Δ*sho1* was 8.80%, compared with 15.55% in the WT (*P* < 0.05), whereas no significant difference was detected under non-stress conditions. Consistent with reduced germination, DCFH-DA fluorescence assays showed higher intracellular ROS levels in Δ*sho1* spores than in WT across the 0–10 mM H_2_O_2_ range (Fig. 4C). These observations indicate that loss of *sho1* is associated with increased ROS accumulation in spores. Because no significant difference in germination was detected at 0 mM H_2_O_2_, elevated basal ROS alone was not sufficient to inhibit germination under non-stress conditions, whereas the germination defect became evident under external oxidative challenge (40). To further assess whether Sho1 affected HOG-associated oxidative signaling, qPCR analysis was performed after 1 h exposure to 0, 6, and 10 mM H_2_O_2_ (Fig. 4D). Under non-stress conditions (0 mM H_2_O_2_), Δ*sho1* showed significantly lower expression of *hog1*, *sod1*, *skn7*, and *gpxr*, which were approximately 0.53-, 0.21-, 0.54-, and 0.38-fold of the WT levels, respectively, whereas *cat2* increased to approximately 1.62-fold of the WT level. In contrast, *sln1* and *trxt* did not differ significantly between the two strains. This basal expression pattern suggests that deletion of *sho1* altered the oxidative-stress preparedness of DM1 even before H_2_O_2_ treatment. Under 6 mM H_2_O_2_, Δ*sho1* exhibited broad transcriptional induction, with *sln1*, *hog1*, *sod1*, *skn7*, *cat2*, *gpxr*, and *trxt* reaching approximately 1.68-, 2.16-, 2.71-, 1.66-, 1.39-, 2.63-, and 1.40-fold of the WT levels, respectively. At 10 mM H_2_O_2_, the strongest increases were observed for *gpxr* and *trxt*, which reached approximately 5.07- and 8.60-fold of the WT levels, while *sln1* and *sod1* remained elevated to approximately 1.65- and 1.89-fold, respectively; by contrast, *hog1*, *skn7*, and *cat2* no longer showed significant differences. Full qPCR bar plots for all tested genes are provided as supplemental raw data (Fig. S3B through D). Together, these concentration-dependent transcriptional changes indicate that deletion of *sho1* altered both the basal redox-state program and the pattern of oxidative-stress-induced transcriptional compensation. Combined with the increased ROS accumulation and reduced spore germination in Δ*sho1*, these results support the conclusion that Sho1 contributes to oxidative-stress resistance and spore viability by helping maintain redox homeostasis in *A. sydowii* DM1.

### Sho1 deletion is associated with altered secondary metabolite profiles and antibacterial activity

**Fig 5:**
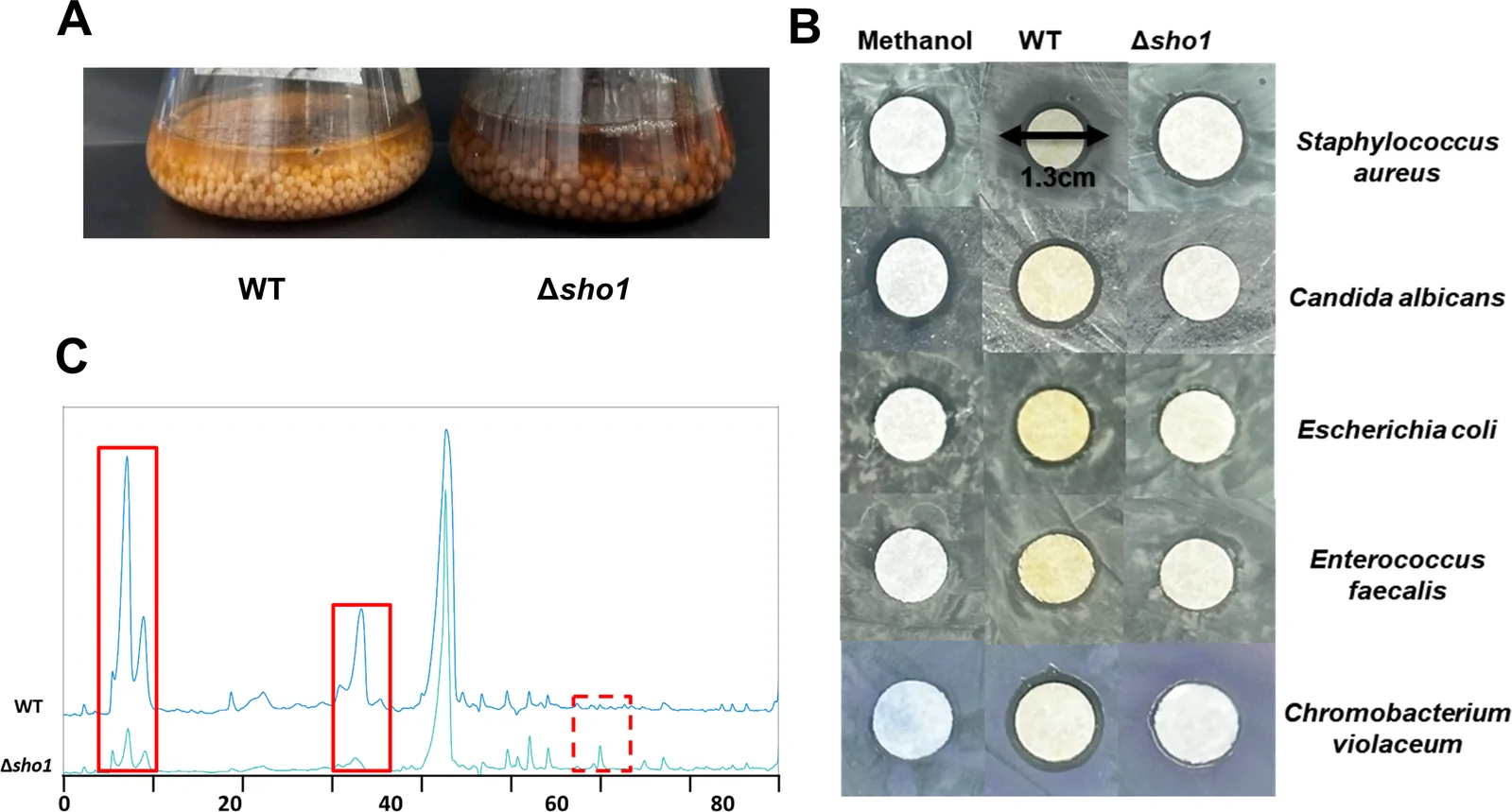
Sho1 deletion is associated with altered antibacterial activity and secondary metabolite profiles in *Aspergillus sydowii* DM1. (**A**) Culture supernatants of WT and Δ*sho1* after shaking in PDB (28°C, 180 rpm) for 10 days. (**B**) Antibacterial activity of ethyl acetate crude extracts from WT and Δ*sho1*, assessed by the disk diffusion assay. (**C**) HPLC chromatograms of ethyl acetate extracts from WT and Δ*sho1*.

Because loss of Sho1 altered oxidative-stress responses and redox homeostasis in DM1, we examined whether it was also associated with changes in secondary metabolite profiles and antibacterial activity. After cultivation in PDB at 28°C with shaking for 10 days, secondary metabolites (SMs) were extracted using ethyl acetate and subjected to antibacterial assays and HPLC analysis. Clear differences in the appearance of the culture supernatants were observed: the WT exhibited a pale yellow coloration, whereas Δ*sho1* displayed a deep red color, suggesting possible alterations in SM composition (Fig. 5A). Antibacterial activity was assessed using a disk diffusion assay against five indicator microorganisms. The crude extract from the WT showed inhibitory activity against *Staphylococcus aureus*, with an inhibition zone diameter of 1.3 cm, whereas no obvious inhibition was detected for the Δ*sho1* extract (Fig. 5B). Neither extract exhibited inhibitory activity against the remaining four indicator strains. HPLC analysis also revealed changes in the metabolite profile of Δ*sho1* compared with the WT, including a reduction in two major peaks and an increase in one peak (Fig. 5C). Together, these findings indicate that loss of *sho1* was accompanied by altered secondary metabolite profiles and reduced antibacterial activity, although the specific compounds and the underlying regulatory links remain to be identified.

### Core role of Sho1 in adaptation to HHP: coordinating early stress response and later recovery

**Fig 6:**
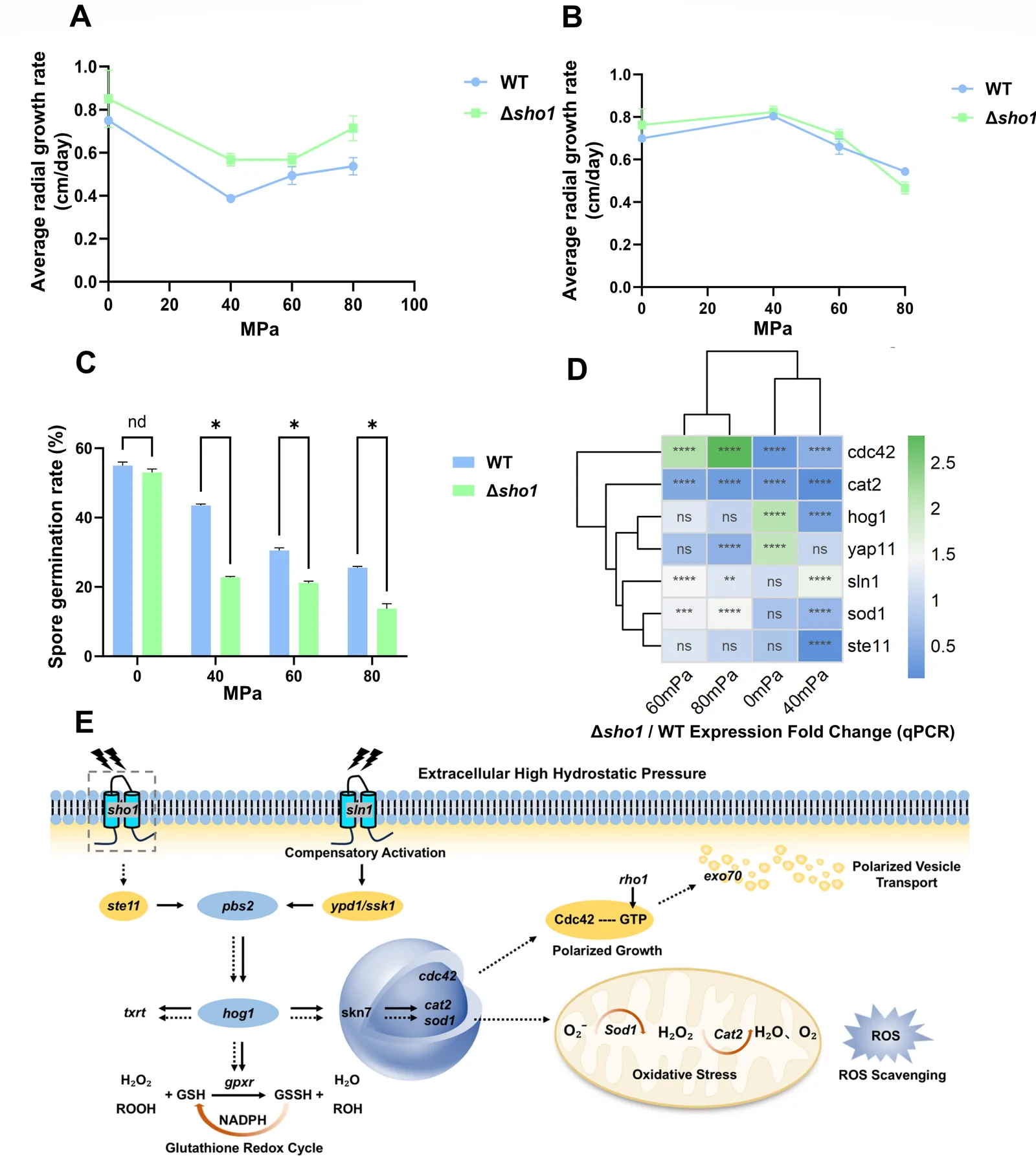
Sho1 is associated with HHP responses during recovery, germination, and pressure-responsive signaling. (**A**) Average radial growth rates of WT and Δ*sho1* during the early recovery phase (days 2–4) after 3 days of HHP treatment. (**B**) Average radial growth rates of WT and Δ*sho1* during the late recovery phase (days 4–6) after 3 days of HHP treatment. (**C**) Spore germination rates of WT and Δ*sho1* after HHP treatment. (**D**) Heat map of qPCR-based relative expression of *hog1*, *sln1*, *ste11*, *yap11*, *cat2*, *sod1*, and *cdc42* under different pressure conditions. (**E**) Proposed Sho1–HOG signaling model under HHP in *Aspergillus sydowii* DM1. The model summarizes Sho1-dependent signaling in WT and the putative compensatory response associated with Sln1 in Δ*sho1*, based on the overall phenotype and qPCR trends. Significance labels indicate comparisons between WT and Δ*sho1* under the same pressure condition: *, *P* < 0.05; **, *P* < 0.01; ***, *P* < 0.001; ****, *P* < 0.0001.

To investigate the role of *sho1* in adaptation to HHP, we analyzed the growth, spore germination, and intracellular ROS accumulation of WT and Δ*sho1* after exposure to different pressure levels (0, 40, 60, and 80 MPa) for 3 days. After pressure treatment, spores were transferred to PDA plates and allowed to recover for 6 days. Because WT and Δ*sho1* already differed under the 0 MPa recovery control, absolute colony diameter after recovery was not interpreted as a purely pressure-specific effect. Instead, we focused on how post-pressure recovery dynamics changed with increasing pressure within each strain. During the early stimulation phase (days 2–4), the growth rate of the WT decreased progressively with increasing pressure, whereas the decline in Δ*sho1* was less pronounced (Fig. 6A). This pattern indicates that pressure did not eliminate the intrinsic growth difference between the two strains, but modified the extent to which early post-pressure growth was reduced. In contrast, during the later recovery phase (days 4–6), the apparent early advantage of Δ*sho1* diminished under extreme pressure, with its recovery rate at 80 MPa (0.46 cm/day) falling below that of the WT (0.54 cm/day) (Fig. 6B). Thus, the pressure effect was more evident in the shift of recovery trajectory across phases than in the absolute colony size difference alone. The germination rates of Δ*sho1* were consistently lower than those of the WT at all pressure levels (Fig. 6C); this difference was particularly evident at 80 MPa (13.7% in Δ*sho1* vs 25.6% in the WT, *P* < 0.05). Consistent with reduced germination, intracellular ROS accumulation in Δ*sho1* spores was significantly higher than in the WT under all pressure conditions (Fig. S4A), with the greatest difference observed at 80 MPa (529 in Δ*sho1* vs 329 in the WT, *P* < 0.05), consistent with greater oxidative stress in Δ*sho1* after HHP treatment. Expression of representative HOG-associated signaling and stress-response genes under HHP was further analyzed by qPCR (Fig. 6D). These included *sln1* and *ste11* as upstream pathway components, *hog1* as the core MAPK, and *yap11*, *cat2*, *sod1*, and *cdc42* as pathway-associated readouts related to oxidative defense and cellular recovery. Under ambient pressure (0 MPa), *hog1* and *yap11* were upregulated in Δ*sho1*, whereas *cat2* and *cdc42* were downregulated relative to the WT. At 40 MPa, *sln1* was upregulated in Δ*sho1*, while *hog1* and *ste11* were reduced, accompanied by further reduction of *cat2*. At 60 and 80 MPa, sustained upregulation of *sln1* and *cdc42* was observed in Δ*sho1*, whereas *yap11* and *cat2* were significantly suppressed at 80 MPa (Fig. 6D).

Based on the combined recovery, germination, ROS, and qPCR patterns, we propose a working model for Sho1-associated pressure responses in *Aspergillus sydowii* DM1 (Fig. 6E). In this model, Sho1 is interpreted as contributing to pressure-responsive signaling and post-stress adaptation, whereas transcriptional induction of *sln1* in Δ*sho1* may reflect a partial compensatory response. Because the expression difference of *hog1* between WT and Δ*sho1* was most evident at 40 MPa rather than across all pressure levels, Fig. 6E should be regarded as an interpretation based on the overall phenotype and expression trends, rather than direct proof of a single branch-specific signaling route. Together, these results support the view that Sho1 contributes to germination, redox balance, and recovery under HHP, while the detailed pathway architecture under pressure remains to be further resolved.

### *Aspergillus sydowii* strains from distinct ecological niches exhibit divergent adaptation strategies to HHP

**Fig 7:**
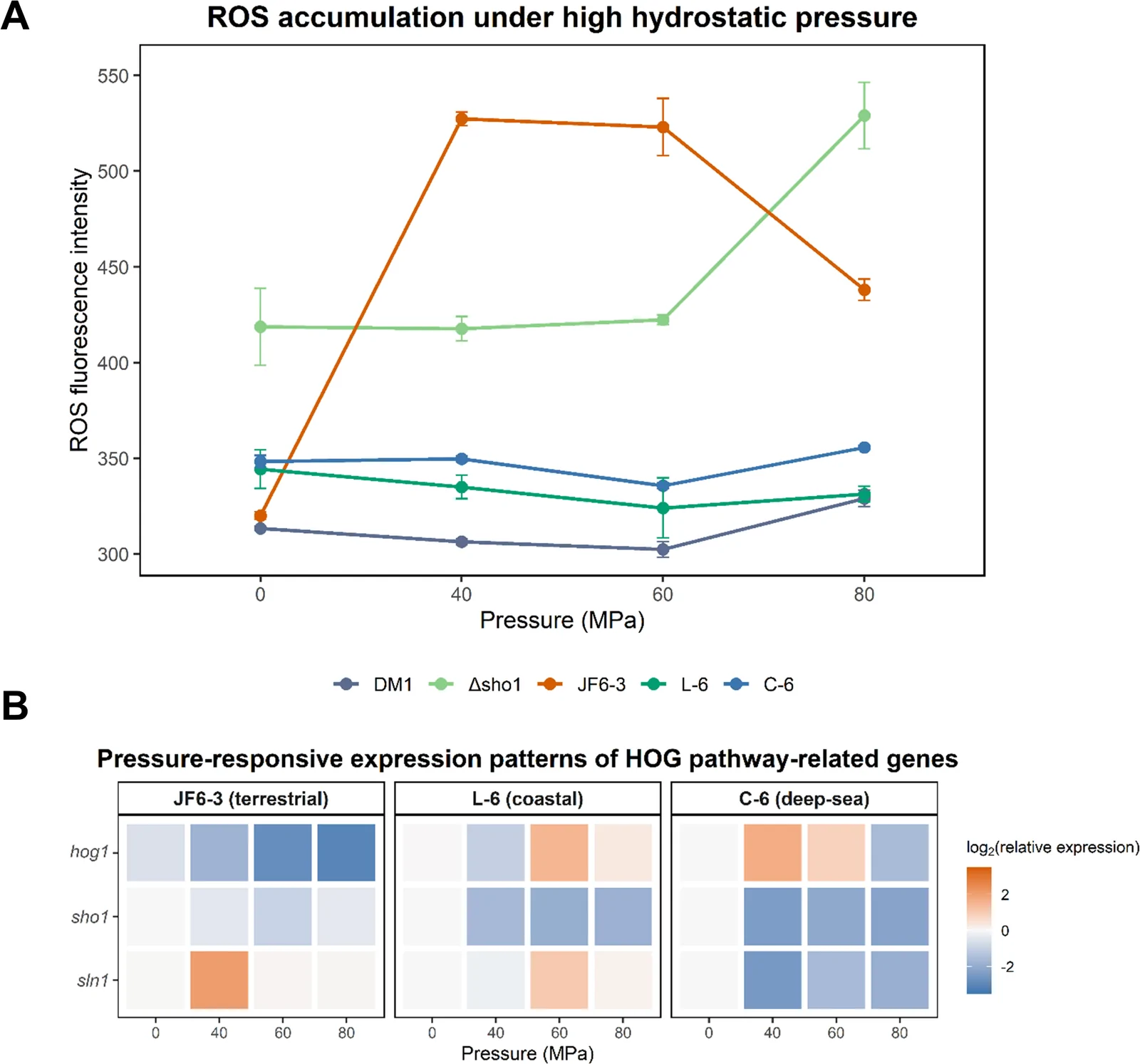
Pressure-associated ROS accumulation and HOG-related expression patterns in *Aspergillus sydowii* strains from different ecological niches. (**A**) Intracellular ROS levels in spores of *A. sydowii* strains from distinct habitats (JF6-3, L-6, C-6, and DM1) and the Δ*sho1* after 3 days of HHP (0, 40, 60, and 80 MPa) treatment. (**B**) Pressure-responsive expression patterns of HOG pathway-related genes in *A. sydowii* strains from different ecological niches. Three mini-heatmaps summarize the relative expression patterns of *sln1*, *sho1*, and *hog1* in the terrestrial strain JF6-3, the shallow-sea strain L-6, and the deep-sea strain C-6 after exposure to 0, 40, 60, and 80 MPa for 3 days. For each strain, expression levels at 0 MPa were used as the baseline. Colors represent the mean log_2_ (relative expression) values from three biological replicates, with warm colors indicating upregulation and cool colors indicating downregulation under pressure.

To explore pressure-associated responses among *Aspergillus sydowii* strains from different ecological niches, we compared intracellular ROS accumulation after HHP treatment among the terrestrial strain JF6-3, the shallow-sea strain L-6, the deep-sea strain C-6, as well as the hadal strain DM1 and the Δ*sho1*. After 3 days of exposure to pressures ranging from 0 to 80 MPa, intracellular ROS accumulation in spores was measured (Fig. 7A). Similar to DM1, the shallow-sea strain L-6 and the deep-sea strain C-6 maintained relatively stable ROS levels across the tested pressure range. In contrast, the Δ*sho1* showed consistently elevated ROS levels and a further increase at 80 MPa, whereas the terrestrial strain JF6-3 displayed pronounced ROS accumulation at 40 and 60 MPa and remained above its baseline level at 80 MPa. These observations suggest that pressure-associated ROS responses differ among strains from distinct habitats, and that loss of *sho1* is associated with a response pattern more similar to that of the terrestrial strain under HHP.

Expression patterns of *sln1*, *sho1*, and *hog1* were determined in JF6-3, L-6, and C-6 (Fig. 7B). In JF6-3, *sln1* showed a transient increase at 40 MPa, whereas *sho1* and *hog1* remained reduced under pressure. In L-6, *sho1* remained low across pressure conditions, while *sln1* and *hog1* showed increased expression at 60 MPa. In C-6, *hog1* was strongly induced at 40 and 60 MPa, whereas *sln1* and *sho1* remained comparatively low. Together, these results suggest that *A. sydowii* strains from different ecological niches exhibit distinct HOG-associated expression patterns under HHP. In combination with the ROS data, the deep-sea strain C-6 and the hadal wild-type strain DM1 showed comparatively stable pressure-associated responses, the shallow-sea strain L-6 displayed an intermediate pattern, whereas the terrestrial strain JF6-3 and the hadal Δ*sho1* exhibited less coordinated responses.

## DISCUSSION

Deep-sea and hadal fungi are increasingly recognized for their ecological significance and biotechnological potential (40, 41); however, the mechanistic understanding of their adaptation to HHP remains limited, largely because functional genetic evidence from cultivable isolates is still scarce. In this study, we provide genetic evidence that an upstream HOG pathway-associated sensor contributes to multiple adaptive traits in the hadal fungus *Aspergillus sydowii* DM1. In our previous work on the hadal fungus *Aspergillus sydowii* DM1, the HOG–MAPK pathway was implicated in pressure-associated stress responses (14), supporting the importance of this conserved signaling system in deep-sea adaptation. Nevertheless, research has largely focused on middle and downstream components, and the functional differentiation of upstream sensing modules remains poorly resolved (13, 42). Within the upstream branches of the HOG pathway, Sho1 is a membrane-localized sensor that, together with Sln1, can function in a context-dependent and partially cooperative manner in model fungi (26, 43, 44). Importantly, DM1 provides a suitable system for this question because it combines clear habitat relevance to hadal high-pressure conditions with an established genetic manipulation platform, allowing direct functional testing of upstream HOG-associated components in a deep-sea fungal background. Here, by generating a *sho1* deletion mutant in DM1 using CRISPR–Cas9, we provide genetic evidence that an upstream HOG-associated sensor contributes to fungal development, cell wall integrity, oxidative homeostasis, secondary metabolism production, and, importantly, is involved in HHP adaptation. Thus, the significance of studying Sho1 in DM1 lies not simply in re-examining a conserved sensor but in determining whether this upstream module contributes to stress coordination under ecologically relevant pressure-associated conditions in a hadal-derived filamentous fungus. In particular, the combined transcriptional analysis of representative HOG-associated genes, including *sln1*, *ste11*, and *hog1*, together with pathway-associated readouts linked to oxidative defense, development, and polarity, provided a clearer framework for interpreting how *sho1* deletion reshaped stress-responsive signaling in DM1 across different experimental conditions. These findings extend current understanding of pressure-related stress signaling in filamentous fungi and help bridge the gap between correlative omics observations and causal pathway function in hadal systems. An additional point that requires clarification is that, under 0 mM H_2_O_2_, Δ*sho1* spores displayed significantly higher intracellular ROS levels than WT spores, whereas no significant difference in spore germination was detected under the same condition. This suggests that elevated basal ROS does not necessarily translate immediately into impaired germination. One possible explanation is that the higher ROS level in Δ*sho1* under non-stress conditions reflects altered basal redox homeostasis rather than acute oxidative damage sufficient to block developmental initiation. In support of this interpretation, the qPCR data at 0 mM H_2_O_2_ showed reduced expression of several oxidative-stress-related genes in Δ*sho1*, including *hog1*, *sod1*, *skn7*, and *gpxr*, whereas *cat2* was increased, suggesting that loss of *sho1* reshaped the basal antioxidant program and may have triggered only partial compensatory regulation. Under these conditions, the mutant may still retain enough physiological capacity to initiate germination, but the elevated ROS background likely lowers its redox buffering capacity. Once additional oxidative stress is imposed, this altered basal state may become insufficient, leading to the more pronounced ROS accumulation and reduced germination observed in Δ*sho1* under H_2_O_2_ treatment. HHP is the defining physical constraint of hadal ecosystems, and recent trench-scale surveys such as the Mariana Trench Environment and Ecology Research (MEER) project have highlighted antioxidation-related functions as recurrent features associated with hadal adaptation (45). However, mechanistic validation of specific adaptive modules in culturable eukaryotic microorganisms remains limited (46). In this context, our results suggest that Sho1 is not simply a positive determinant of pressure tolerance, but may instead help coordinate a trade-off between immediate growth restraint and effective post-stress recovery. Specifically, the Δ*sho1* showed weaker early growth inhibition after pressure treatment, yet this apparent short-term advantage was accompanied by reduced spore germination, increased ROS accumulation, and poorer late recovery under extreme pressure. Importantly, because WT and Δ*sho1* already differed under the 0 MPa recovery control, this interpretation does not rely on endpoint colony diameter alone, but rather on the pressure-dependent shift in recovery dynamics across phases together with the accompanying germination and ROS phenotypes. Even if active germination and hyphal extension are strongly restricted during pressurization, release from pressure does not necessarily restore spores to equivalent physiological states; latent differences in redox balance, cellular damage, and developmental readiness may still lead to divergent post-pressure recovery trajectories. One possible interpretation is that Sho1-dependent HOG signaling normally imposes a transient protective brake on proliferation during and immediately after pressure stress, while simultaneously supporting oxidative homeostasis and the recovery competence required for subsequent development (47). When this branch is absent, early colony expansion may appear less restricted, but the mutant may pay for this with impaired redox control and reduced capacity to resume robust growth. This interpretation is broadly consistent with the canonical role of Hog1 in stress adaptation, where HOG signaling is linked to transient stress-induced cell-cycle delay (42)(47), and with previous work in *Aspergillus fumigatus* showing that loss of *sho1* delays conidial germination and compromises oxidant adaptation (48). At the same time, our data also extend these earlier observations by suggesting that, under hydrostatic pressure, release from Sho1-associated growth restraint can be uncoupled from true stress resilience. Thus, rather than acting only as an upstream stress sensor, Sho1 may contribute to balancing growth, oxidative defense, and recovery timing under HHP. Future work should test this idea more directly by examining pressure-induced Hog1 activation dynamics, complementation of Δ*sho1*, and the temporal relationship between stress signaling, cell-cycle progression, and germination during pressure recovery.

An important point in interpreting our data is that colony expansion, spore germination, and ROS accumulation represent different biological dimensions of stress adaptation and therefore should not be expected to change in parallel. Colony diameter mainly reflects radial hyphal expansion after inoculation or recovery, whereas spore germination reflects the capacity of dormant propagules to initiate development, and ROS accumulation reflects intracellular redox burden. In this context, the apparently faster radial growth of Δ*sho1* should not be interpreted as stronger stress tolerance. Instead, our data suggest that loss of Sho1 partially releases growth restraint under some stress conditions, allowing faster colony expansion, but at the cost of impaired redox homeostasis and reduced developmental competence. This interpretation is supported by the reduced germination and elevated ROS levels of Δ*sho1* under oxidative stress and HHP, as well as by the altered expression of HOG-associated and stress-responsive genes. Thus, the seemingly contradictory phenotype of Δ*sho1* can be explained as a partial uncoupling between hyphal expansion and stress-protective regulation. In this framework, Sho1 appears to function less as a simple determinant of growth inhibition and more as a coordinator that links environmental sensing to developmental timing, oxidative homeostasis, and recovery capacity in *Aspergillus sydowii* DM1. The secondary metabolite analysis should also be interpreted cautiously. In this study, HPLC peak profiles and antibacterial assays were used to compare overall metabolite-associated phenotypes between WT and Δ*sho1*, but the specific compounds responsible for the altered peaks or antibacterial activity were not identified. Therefore, these data indicate that *sho1* deletion is associated with changes in secondary metabolite profiles and antibacterial activity, but they do not allow assignment of specific metabolites. Future LC-MS- or NMR-based analyses will be required to identify the compounds affected by Sho1-associated regulation.

Sho1 has been extensively studied as an upstream input module of the HOG–MAPK network in fungi (42), and its biological relevance extends beyond osmosensing to broader coordination of cell wall homeostasis, oxidative defense, and developmental regulation in a species- and context-dependent manner (49, 50). This functional diversity highlights Sho1 as a strategic node for understanding how conserved signaling architectures are rewired across fungal lineages and ecological niches (30, 48, 51). Yet, despite the centrality of Sho1 in canonical stress signaling, its potential involvement in responding to a purely physical cue such as hydrostatic pressure has been largely overlooked (52, 53), especially in filamentous fungi from deep-sea environments (13, 14). Our findings extend the current Sho1 framework by suggesting that a membrane-localized HOG-associated sensor can contribute to pressure-responsive signaling outputs in a hadal filamentous fungus, thereby linking upstream sensing to downstream stress defense in a pressure-relevant context. Conceptually, this supports the idea that deep-sea eukaryotes may achieve piezotolerance not by inventing entirely new pathways, but by repurposing conserved membrane-associated sensing modules and their coupling to redox and cellular integrity programs (42, 54). More broadly, positioning Sho1 at the interface of environmental perception and pathway remodeling provides a mechanistic entry point for dissecting eukaryotic pressure sensing, and encourages future work to resolve how membrane properties and sensor–scaffold interactions translate hydrostatic pressure into regulated MAPK outputs (23, 29, 55).

The niche-comparative analysis across *A. sydowii* strains from terrestrial, shallow-sea, deep-sea, and hadal origins provides an informative first glimpse into how pressure-associated responses may differ along an environmental gradient; however, this component should be interpreted as exploratory rather than definitive evidence for a linear evolutionary trajectory (45, 56). A key uncertainty is whether the observed divergence primarily reflects niche-driven tuning of signaling logic (e.g., upstream sensor usage and downstream redox control) or is partly attributable to strain-specific baseline physiology, such as differences in sporulation, intrinsic antioxidant capacity, and growth dynamics that can influence ROS and germination readouts (57–59). In particular, the terrestrial strain JF6-3 showed a distinct ROS accumulation pattern compared with the marine-derived strains. Its pronounced ROS increase at 40 and 60 MPa may reflect a weaker capacity to buffer pressure-induced oxidative imbalance, possibly because this strain has not been exposed to long-term selection under deep-sea or high-pressure conditions. This interpretation is also consistent with its HOG-associated expression pattern, in which *sho1* and *hog1* did not show coordinated induction under pressure, whereas the deep-sea strain C-6 showed stronger *hog1* induction and relatively stable ROS levels. Therefore, the divergent ROS trend of JF6-3 likely reflects both its terrestrial ecological background and strain-specific stress-response capacity, rather than a universal response of *A. sydowii* to HHP. In addition, because the current comparison relies mainly on phenotypic and transcriptional outputs under defined pressure treatments, it cannot fully resolve causality or distinguish direct pathway control from broader stress-network convergence (60–62). Another limitation of the present study is that a stable SHO1-complemented strain was not available for parallel validation. Although we have made repeated attempts to construct a SHO1-complemented strain, a stable and fully validated complemented strain has not yet been obtained, suggesting that efficient genetic manipulation in DM1 may require further strain-specific optimization of transformation conditions and selection markers, as reported for other marine-derived fungi (63, 64). Therefore, although three independent mutants, PCR/sequencing verification, and the consistency of multiple stress-associated readouts support an important role of Sho1 in stress adaptation, the present conclusions should still be interpreted as strong genetic evidence from the deletion mutant rather than definitive complementation-based proof of causality. To strengthen this framework in a feasible and stepwise manner, future work should incorporate pathway-level activity readouts under pressure, such as time-resolved Hog1 activation dynamics (e.g., phosphorylation) alongside osmotic controls (13, 65), establish minimal genetic causality through *sho1* complementation and targeted perturbation of alternative upstream modules where possible (13, 66), and improve cross-strain comparability by standardizing baseline measurements and presenting pressure-induced changes relative to each strain’s 0 MPa state (13, 14). Together, these focused steps would allow a more rigorous evaluation of which features represent habitat-associated specialization to high pressure and which reflect broader intraspecies diversity in stress responses. Nevertheless, the present cross-niche comparison should still be regarded as an initial framework rather than a definitive model, and many mechanistic and evolutionary questions remain open for further investigation.

## SUPPLEMENTAL MATERIAL

Supplemental material aem.00898-26-s0001.docx Fig. S1 to S4; Tables S1 and S2.

ASM does not own the copyrights to Supplemental Material that may be linked to, or accessed through, an article. The authors have granted ASM a non-exclusive, world-wide license to publish the Supplemental Material files. Please contact the corresponding author directly for reuse.
