# Supplementary material for: Insight into the role of Sho1 in high-hydrostatic pressure and oxidative stress adaptation of the hadal fungus Aspergillus sydowii DM1
Source: Appl Environ Microbiol. 2026 Jul 16;92(8):e00898-26. doi: 10.1128/aem.00898-26 (PMC13488358; doi:10.1128/aem.00898-26)
Supplement: Supplementary file 1 [file aem.00898-26-s0001.docx]

**Insight into the role of Sho1 in high-hydrostatic pressure and oxidative stress adaptation of the hadal fungus *Aspergillus sydowii* DM1**

Hongfu Lai , Guangzhao Hu, Yukun Cui , and Xi Yu^*1^

**Running head:** Sho1-mediated high-pressure adaptation in hadal fungi

**Address**:	**^1^**Shanghai Engineering Research Center of Hadal Science and Technology, College of Oceanography and Ecological Science, Shanghai Ocean University, Shanghai, 201306, China;

*Corresponding author:	XY (x[yu@shou.edu.cn](mailto:yu@shou.edu.cn)，orcid:http://orcid.org/0000-0003-4243-6016);

**Key words:** Deep-sea fungi; High hydrostatic pressure; Sho1; HOG-MAPK pathway; Oxidative stress; CRISPR-Cas9

**Fig. S1**

**Supplementary Figure S1 Validation and representative morphology of independent Δ*sho1* transformants constructed by CRISPR–Cas9.** (A) Schematic diagram of CRISPR–Cas9-mediated deletion of the 1,077-bp *sho1* coding region using dual sgRNAs and primer positions for PCR identification. The expected PCR fragment was approximately 1.6 kb in WT and 0.7 kb in Δ*sho1*. (B) Screening of transformants on PDA plates containing hygromycin B. The handwritten marks on the plates indicate internal experimental labels, including the control group, the sgRNA-transformed group, and plate identifiers, and have no additional biological meaning. (C) PCR verification of WT and three representative Δ*sho1* transformants using primers flanking the *sho1* locus. (D) Sequence alignment of PCR products confirming deletion of the 1,077-bp *sho1* coding region with intact flanking sequences in the representative Δ*sho1* strain. (E) Representative colony morphology of WT and three independent Δ*sho1* transformants on PDA supplemented with 1 M NaCl.

**F****ig. S2**

**C**

**B**

**A**

**D**

**Supplementary Figure 2 Full qPCR bar plots corresponding to the salinity-responsive transcriptional changes summarized in Fig. 4.**(A) Relative expression of HOG pathway-related genes (*sln1, hog1, ste11, brlA, ssk1,* and *skn7*) in WT and Δ*sho1* under 0.5 M NaCl.(B) Relative expression of HOG pathway-related genes in WT and Δ*sho1* under 1.0 M NaCl.(C) Relative expression of *sln1* and other core HOG components in WT and Δ*sho1* under 2.0 M NaCl.(D) Relative expression of polarity establishment- and vesicle trafficking-related genes (*rho1, rho3, cdc42, exo70,* and *cla4*) in WT and Δ*sho1* under 3.0 M NaCl.

**Fig. S3**

**B**

**A**

**C**

**Fig. S3**

**Supplementary Figure 3 Additional quantitative and full qPCR data for oxidative-stress assays in WT and Δ*sho1*.** (A) Colony diameters of WT and Δ*sho1* strains under H₂O₂ stress (0–8 mM) after 7 days. At 10 mM H₂O₂, neither strain formed measurable colonies and this condition was therefore not included in the diameter analysis. (B–D) Relative expression levels of oxidative stress-related genes (*sln1, hog1, sod1, skn7, cat2, gpxr,* and *trxt*) in WT and Δ*sho1* strains after 1 h treatment with 0 mM (B), 6 mM (C), and 10 mM (D) H₂O₂, as determined by qRT-PCR.

**D**

**Fig. S4**

**A**

**B**

**Supplementary Figure 4 Additional quantitative data for high-hydrostatic-pressure responses in WT and Δ*sho1*.** (A) Intracellular ROS levels in spores after HHP. (B) Colony diameters of WT and Δ*sho1* after 3 d high hydrostatic pressure treatment (0, 40, 60, and 80 MPa), followed by transfer to PDA and recovery incubation for 6 d under atmospheric pressure.

**Table. S1**

**Supplementary Table 1** Information on primers used in qRT-PCR.

| Gene | Primer name | sequence (5'-3') | Purpose |
| --- | --- | --- | --- |
| *sho1* | sho1F | CCGCTCAGGAGTTGCTAAGA | RT-qPCR |
|  | sho1R | CGCACCGAGTCTGACATCA |  |
| *sln1* | sln1F | GCAATTCTCAGGAAGCAACTCA | RT-qPCR |
|  | sln1R | AGCATCACAATCATCGCAGAC |  |
| *ste11* | ste11F | ATTCATCTGCGTCAGGTCTCT | RT-qPCR |
|  | ste11R | TGGCGGAGGTCACATACATC |  |
| *hog1* | hog1F | ACTCTTCCTTGGTCTTCGTCTT | RT-qPCR |
|  | hog1R | AGTCCGTGTATCTGCGAGTC |  |
| *cat2* | cat2F | GGCTTCTCTGTCAAGTTCTTCA | RT-qPCR |
|  | cat2R | ACTCGGCGGATGGAATCTG |  |
| *sod1* | sod1F | GGTCAAGGCTGTTGCTGTC | RT-qPCR |
|  | sod1R | CGAACTGGTGGATGTGGAAG |  |
| *gpxr* | gpxrF | CGGATTCACGCCTCAATTCAA | RT-qPCR |
|  | gpxrR | CGCCTTGTCGCCATTAACC |  |
| *trxt* | trxtF | CTTACCACGACCACCGATGT | RT-qPCR |
|  | trxtR | CTGCCAGTAGACCTCCTCAC |  |
| *yap11* | yap11F | GAACGATGCCACACCGATAC | RT-qPCR |
|  | yap11R | AGAGTCTGACGATGAAGATGGA |  |
| *skn7* | skn7F | GCGTGTCATCTCATTCGTCAA | RT-qPCR |
|  | skn7R | GAATTGTTGCTGGCGTTGTG |  |
| *rho1* | rho1F | TTCTTCGTCAACCGCAATGG | RT-qPCR |
|  | rho1R | CACAACATCTTCGCCTGTATCA |  |
| *cdc42* | cdc42F | GCCTATCACGCCAGAGCAA | RT-qPCR |
|  | cdc42R | CCACCACCACTACCACCATTA |  |
| *exo70* | exo70F | ACTACATCCGCTCCAATCTCAT | RT-qPCR |
|  | exo70R | AGACGCAAGAGGCTTAGAGTAT |  |
| GAPDH | GAPDHF | GATCGTCAACGGCAAGAAGAT | reference gene |
|  | GAPDHR | AGCGGAAGGAGCAGAGATG |  |

**Table. S2**

**Supplementary Table 2** Sequence similarity and conserved-domain annotation of the *sho1* sequence in *Aspergillus sydowii* DM1.

| Analysis | Species / Domain | Annotation / Description | Query cover | Identity | E-value | Accession / Domain ID |
| --- | --- | --- | --- | --- | --- | --- |
| BLASTN | *Aspergillus sydowii* CBS 593.65  sydowii CBS 593.65 | osmosensor SHO1 (*sho1*), partial mRNA | 94% | 93.19% | 0.0 | XM_040844956.1 |
| BLASTN | *Aspergillus* puulaauensis | osmosensor SHO1 (*sho1*), partial mRNA | 94% | 88.27% | 0.0 | XM_041696437.1 |
| BLASTN | *Aspergillus* versicolor CBS 583.65 | osmosensor SHO1 (*sho1*), partial mRNA | 83% | 86.96% | 0.0 | XM_040815689.1 |
| BLASTN | *Aspergillus* undulatus | osmosensor SHO1 (*sho1*), mRNA | 89% | 79.84% | 0.0 | XM_073754415.1 |
| BLASTN | *Aspergillus* stella-maris | osmosensor SHO1 (*sho1*), partial mRNA | 88% | 77.20% | 1e-164 | XM_073771278.1 |
| BLASTN | *Aspergillus* nidulans FGSC A4 | osmosensor SHO1 (*sho1*), mRNA | 69% | 82.66% | 1e-145 | XM_675875.2 |
| Conserved domain search | SH3_Sho1p | Src homology 3 domain of high-osmolarity signaling protein Sho1p | — | — | 1.22 × 10^-22 | cd11855 |
|  |  |  |  |  |  |  |
